# Effect of Porcine *Clostridium perfringens* on Intestinal Barrier, Immunity, and Quantitative Analysis of Intestinal Bacterial Communities in Mice

**DOI:** 10.3389/fvets.2022.881878

**Published:** 2022-06-13

**Authors:** Zipeng Jiang, Weifa Su, Chaoyue Wen, Wentao Li, Yu Zhang, Tao Gong, Shuai Du, Xinxia Wang, Zeqing Lu, Mingliang Jin, Yizhen Wang

**Affiliations:** ^1^National Engineering Laboratory of Biological Feed Safety and Pollution Prevention and Control, Zhejiang University, Hangzhou, China; ^2^Key Laboratory of Molecular Nutrition, Ministry of Education, Zhejiang University, Hangzhou, China; ^3^Key Laboratory of Animal Nutrition and Feed, Ministry of Agriculture and Rural Affairs, Zhejiang University, Hangzhou, China; ^4^Key Laboratory of Animal Nutrition and Feed Nutrition of Zhejiang Province, Zhejiang University, Hangzhou, China; ^5^College of Animal Science, Institute of Feed Science, Zhejiang University, Hangzhou, China

**Keywords:** *Clostridium perfringens*, mice model, intestinal barrier, immune status, apoptosis

## Abstract

*Clostridium perfringens* (*C. perfringens*) is one of the main pathogens which can cause a range of histotoxic and enteric diseases in humans or animals (pigs, or broilers). The Centers for Disease Control and Prevention (CDC) estimates these bacteria cause nearly 1 million illnesses in the United States every year. For animal husbandry, necrotizing enteritis caused by *C. perfringens* can cost the global livestock industry between $2 billion and $6 billion per yea*r*. *C. perfringens*-infected animals can be isolated for its identification and pathology. A suitable animal model is one of the essential conditions for studying the disease pathogenesis. In previous studies, mice have been used as subjects for a variety of Clostridium perfringens toxicity tests. Thus, this study was designed to build a mouse model infected porcine *C. perfringens* which was isolated from the *C.perfringens*-infected pigs. A total of 32 6-week-old male C57BL/6 mice were randomly divided into four groups. Control group was orally administrated with PBS (200 μL) on day 0. Low group, Medium group, and High group were gavaged with 200 ul of PBS resuspension containing 8.0 × 10^7^ CFU, 4.0 × 10^8^ CFU, and 2.0 × 10^9^ CFU, respectively. We examined growth performance, immune status, intestinal barrier integrity, apoptosis-related genes expression, and copies of *C. perfringens* in mice. The results showed that the growth performance declined and intestinal structure was seriously damaged in High group. Meanwhile, pro-inflammatory factors (IL-1β, TNF-α, and IL-6) were significantly increased (*P* < 0.05) in High group compared to other groups. The tight junctions and pro-apoptosis related genes' expression significantly decreased (*P* < 0.05) in High group, and high dose caused a disruption of intestinal villi integrity and tissue injury in the jejunum of mice. In addition, the enumerations of *C. perfringens, Escherichia coli*, and *Lactobacillus* explained why the gut of High group mice was seriously damaged, because the *C. perfringens* and *Escherichia coli* significantly enriched (*P* < 0.05), and *Lactobacillus* dramatically decreased (*P* < 0.05). Overall, our results provide an experimental and theoretical basis for understanding the pathogenesis and exploring the effects of porcine *C. perfringens* on mice.

## Introduction

*Clostridium perfringens* is a gram-positive, spore-forming, anaerobic, rod-shaped bacterium (1). *C. perfringens* was isolated from a broad range of environments, such as the soil, freshwater sediment, and the gastrointestinal track of human and animals (2). As an opportunistic pathogen [opportunistic pathogen is one that generally does not harm its host but can when the host's resistance is low (3)], *C. perfringens* can cause disease; it causes a range of histotoxic and enteric diseases in humans and animals (4). *C. perfringens* bacteria are one of the most common causes of foodborne illness (food poisoning). The Centers for Disease Control and Prevention (CDC) estimates these bacteria cause nearly 1 million illnesses in the United States every year (5, 6). For animal husbandry, necrotizing enteritis caused by *C. perfringens* can cost the global livestock industry between $2 billion and $6 billion per year (7, 8). *C. perfringens* mainly causes hemorrhagic necrotizing enteritis in piglets, and triggers “sudden death” disease characterized by abdominal bulging in fattening pigs and pregnant sows, with an 100% mortality rate (9). It is necessary to fully explore the prevention and treatment methods for the disease caused by *C. perfringens* in pigs. Therefore, establishing an animal model is the best way to visually study and obtain data on pathological damage.

A suitable animal model is one of the essential conditions for studying the disease pathogenesis (10). Some studies have used intramuscular or intravenous injections to establish mouse models of *C. perfringens* infection (11–13), and others have modeled D and C aeruginosa by inoculation in the duodenum, intragastric inoculation, or by imposing an oral challenge (14–17). These studies focused on results primarily from a toxemia following absorption of toxins from the intestine into the circulation and they did not fully investigate the concrete changes during *C. perfringens* infection. In the present study, we use the mouse model for understanding the pathogenesis of porcine *C. perfringens* by oral gavage. Furthermore, negative effects (weight loss, decreased expression of tight junction proteins, intestinal morphological damage, etc.) of porcine *C. perfringens* in mouse model were evaluated by determining intestinal morphology, immune status, intestinal barriers integrity, apoptosis, and enumerations of pathogens or probiotics. Our study aims to provide a suitable animal model for the research of the prevention of a pathogenic mechanism of porcine *C. perfringens*.

## Materials and Methods

All the procedures were approved by the Institutional Animal Care and Use Committee at Zhejiang University.

### Bacterial Strain Preparation

Our laboratory originally isolated the pathogenic bacterium which induced the death of swine in a farm from Tech-Bank Co., Ltd. This bacterium was extracted by bacteria genomic DNA kit (Tiangen Biotech Co., Ltd. Beijing). Then PCR amplification was performed using 16S rDNA specific primers and PCR products were subject to purification and Sanger sequencing. We exerted the NCBI (Gene Bank database) to detect the species of this pathogen. In this experiment, this porcine *C. perfringens* was cultured anaerobically on tryptose-sulfite-cycloserine (TSC) agar for 18 h at 37°C, and then transferred to a reinforced clostridium medium (RCM) for analysis (anaerobic environment). The bacteria were harvested by centrifugation at 4,000 g for 10 min at 4°C, and washed 3 times with phosphate-buffered saline (PBS) solution. Finally, we obtained 8.0 × 10^7^ to 2.0 × 10^9^ CFU (colony forming units) *C. perfringens*.

### Animals Experiment

**Figure 1:**
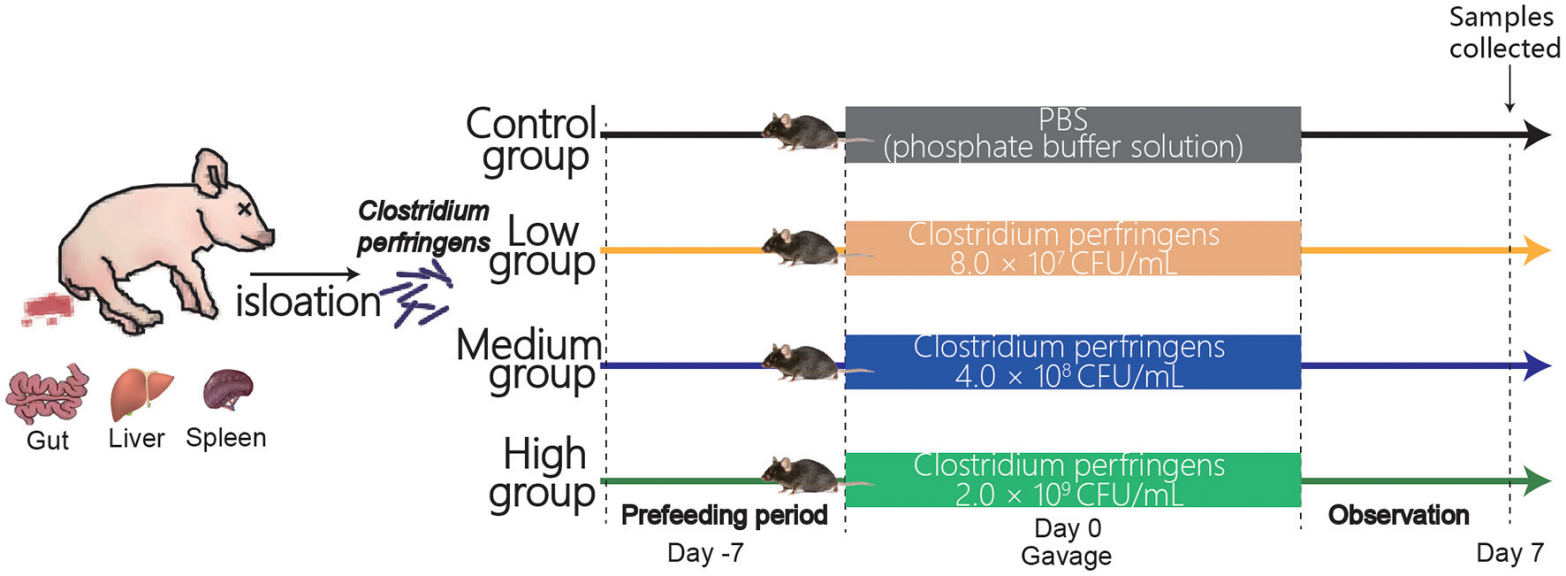
Experimental design and scheme of the animal treatments.

Thirty-two mice (5-week-old male C57BL/6) were purchased from Shanghai Laboratory Animal Co., Ltd. (SLAC, Shanghai, China). All mice were randomly divided into four groups (Figure 1) after 1-week adaptation: Control group, Low group, Medium group, and High group. Mice in Control group were treated orally with 200 μl PBS. Meanwhile, mice in the Low, Medium, and High groups were gavaged with 200 μl of PBS resuspension containing 8.0 × 10^7^ CFU, 4.0 × 10^8^ CFU, and 2.0 × 10^9^ CFU, respectively. Mice were weighed every single day and offered free access to the water and feed during the experimental period. The animal experimental protocol was approved by the Animal Care and Use Committee of Zhejiang University.

### Sample Collection

On day 7, all mice in each group were weighed and sacrificed to collect liver, spleen, colon, blood samples, jejunum, ileum, and fresh digesta in the intestine. The weight of liver and spleen were recorded and was used to calculate the organ index. The colon length was measured by vernier caliper. The blood samples were collected by cardiac puncture and centrifugated at 3, 000 g for 10 min at 4°C, then serum was obtained. The jejunum was washed with cold PBS and prepared for morphology analysis and gene expression determination (−80°C). Simultaneously, the digesta in the intestine were obtained for determining the microbiota enumeration.

### Intestinal Morphology Analysis

The tissues of duodenum, jejunum, and ileum were fixed by 4% paraformaldehyde, then these tissues were excised, embedded in paraffin, sliced, and stained with hematoxylin an eosin (H&E) according to pervious methods (18, 19). Images of paraffin section were observed and obtained with a Lecia DM3000 Microsystem (Leica, Wetzlar, Germany). The villus height and crypt depth were measured by previous studies (19, 20). All paraffin sections were determined from at least 10 well-oriented villus-crypt units by upright fluorescence microscopy using a BX51 microscope (Olympus, Tokyo, Japan).

### Inflammatory Cytokines and Immunoglobulin in Serum and Feces of Mice

Serum parameters, including inflammatory cytokines IL-1β, IL-6, TNF-α and immunoglobulin IgA, IgG, and fecal sIgA, were determined using ELISA kits (Jiangsu Enzyme-Labeled Biological Technology, Jiangsu, China). The protocols were carried out according to the manufacturer's instructions and followed by previous studies (21). Standard 50 μl was added to standard well, then 40 μl sample dilution was added to the to testing sample well, then 10 μl testing sample was added (sample final dilution is 5-fold). To each well, 100 μl HRP-conjugate reagent was added. After closing plate with the closure plate membrane, the samples were incubated for 60 min at 37°C. After uncovering the late membrane, the liquid was discarded, dried by swinging, and washing buffer was added to every well, kept still for 30s and then drained. This was repeated 5 times and then the membranes were pat dry. Chromogen Solution A 50 μl and Chromogen Solution B were added to each well, with light preservation evaded for 15 min at 37°C. Stop Solution of 50 μl was added to each well. The reaction was then stopped (the blue color changed to yellow color). The blank well was taken as zero, and absorbance was read at 450 nm after adding Stop Solution and within 15 min.

### RNA Extraction and Gene Expression Analysis

Total RNA isolation from the tissues was carried out according to previous studies (22, 23), using Trizol reagent (Invitrogen Life Technologies, Carlsbad, CA) according to the manufacturer's instructions. NanoDrop 2000 (Thermo Scientific, Waltham, MA, USA) was used to measure the optical density at 260 to 280 and quantified the purity of the RNA. One microgram of total RNA was reverse transcribed by the reverse transcription kit (Takara Biotechnology Inc., Ostu, Japan) with random primers following the manufacturer's instructions. Subsequently, all the cDNA were obtained. The mRNA expression of *zonula occludens 1* (*ZO-1*), *Occludin, Claudin1, MUC2, p53, Bax, Bcl-2, Caspase-3*, and *Caspase-9* in the jejunum were measured by quantitative real-time PCR (qRT-PCR) analysis. The qRT-PCR assay was conducted on a StepOne Real-Time PCR System (ABI StepOnePlus, Applied Biosystem, Foster City, California) using commercial SYBR-Green PCR-kit (Takara Biotechnology Inc., Japan). Gene-specific primers (Table 1) were used for this process. Finally, the β*-actin* was used as the housekeeping gene, and relative mRNA gene expression were detected by using the 2^−Δ*ΔCt*^ method as previously described (21).

**Table 1:** Primer sequences for q-PCR.

**Gene**	**Forward primer sequence (5^′^−3^′^)**	**Reverse primer sequence (5^′^−3^′^)**	**Accession number**
β-actin	CTAGGCGGACTGTTACTGAGC	CGCCTTCACCGTTCCAGTTT	NM_007393.5
ZO-1	GAAGTTACGTGCGGGAGCAG	GGGACAAAAGTCCGGGAAGC	NM_001163574.1
MUC-2	GCCCACCTCACAAGCAGTAT	GTCATAGCCAGGGGCAAACT	NM_023566.4
Claudin1	TATGACCCCTTGACCCCCAT	AGAGGTTGTTTTCCGGGGAC	NM_016674.4
Occludin	TGAGCACCTTGGGATTCCG	AAAAGGCCTCACGGACATGG	NM_008756.2
p53	GGGCTGAGACACAATCCTCC	CATTGTAGGTGCCAGGGTCC	NM_001127233.1
Bax	CTGGATCCAAGACCAGGGTG	CCTTTCCCCTTCCCCCATTC	NM_007527.3
BCL-2	TGAGTACCTGAACCGGCATC	TTGTGGCCCAGGTATGCAC	NM_009741.5
Caspase-3	GCTTGGAACGGTACGCTAAG	CCACTGACTTGCTCCCATGT	NM_001284409.1
Caspase-9	CACCTTCCCAGGTTGCCAAT	CAAGCCATGAGAGCTTCGGA	NM_001277932.1

### *Clostridium perfringens* Enumeration of Ileum and Cecum

The population of *C. perfringens* in the digesta was detected by absolute qRT-PCR methods, described in previous studies (24–26). The QIAamp DNA Mini Kit (QIAGENLtd., Hilden, Germany) was exerted to isolate the genomic DNA from the ileum and caecum (200 mg of digesta). Extracted DNA was stored at −80°C for further analysis.

Normal PCR amplification was used to produce high concentrations of the target DNA from pure bacterial cultures and standard curves were prepared using it. Primer sequences were designed on the basis of 16s rRNA according to the previous study (Table 2) (28). Competent *Escherichia coli* DH5α (Takara Bio Inc., Japan) was applied to generate plasmid standards. PCR purification kit (Biomed Gene Technologies, Beijing, China) was used to purify PCR products and TA cloning kit (Invitrogen Corporation, Carlsbad, CA, USA) was accessed to clone into pCR™2.1 as per the manufacturer's instruction. Nanodrop 2000 (Thermo Fisher Scientific Inc., Waltham, MA, USA) was exerted to quantify the purified insert-containing plasmids. Then the number of target DNA copies was calculated by the following formula according to Lee et al. (29).


$$\text{DNA }(\text{copy})\text{=}\frac{\text{6}.\text{02 }\times \;{\text{10}}^{\text{23}}\;(\text{copy/mol})\;\times \text{ DNA amount }(\text{g})}{\text{DNA length }\left(\text{dp}\right)\;\times \text{ 660 }(\text{g/mol/dp})}$$

Ten-fold serial dilutions of plasmid DNA method were supported to construct the Standard curve. Finally, the DNA from ileal and cecal samples was determined for absolute qRT-PCR using a StepOne Real-Time PCR System (ABI StepOnePlus, Applied Biosystem, Foster City, California) according to commercial SYBR-Green PCR-kit (Takara Biotechnology Inc., Japan) protocols.

**Table 2:** The sequence of 16s rRNA qRT-PCR primers used to quantify intestinal bacteria.

**Target**	**Primer sequence (5^′^-3^′^)**	**Amplicon size, bp**	**Reference**
*Clostridium*	F: ATGCAAGTCGAGCGAKG	105	(27)
*perfringens*	R: TATGCGGTATTAATCTYCCTTT		
*Lactobacillus*	F: AGCAGTAGGGAATCTTCCA	341	(25)
*subgroup*	R: CACCGCTACACATGGAG		
*Escherichia*	F: GTTAATACCTTTGCTCATTGA	340	(24)
*subgroup*	R: ACCAGGGTATCTAATCCTGT		

### Statistical Analysis

SPSS 20.0 software (SAS Inc., Chicago, IL) was used for analyzing the present study data. One-way ANOVA and Duncan's test were used to determine the difference among groups. Data were expressed as the mean ± standard deviation (SD). *P* < 0.05 was considered statistically significant. GraphPad Prism 8 (San Diego, CA, USA) was used to generate bar plots.

## Results

### Isolation of *Clostridium perfringens* and Construction of Standard Curve

**Figure 2:**
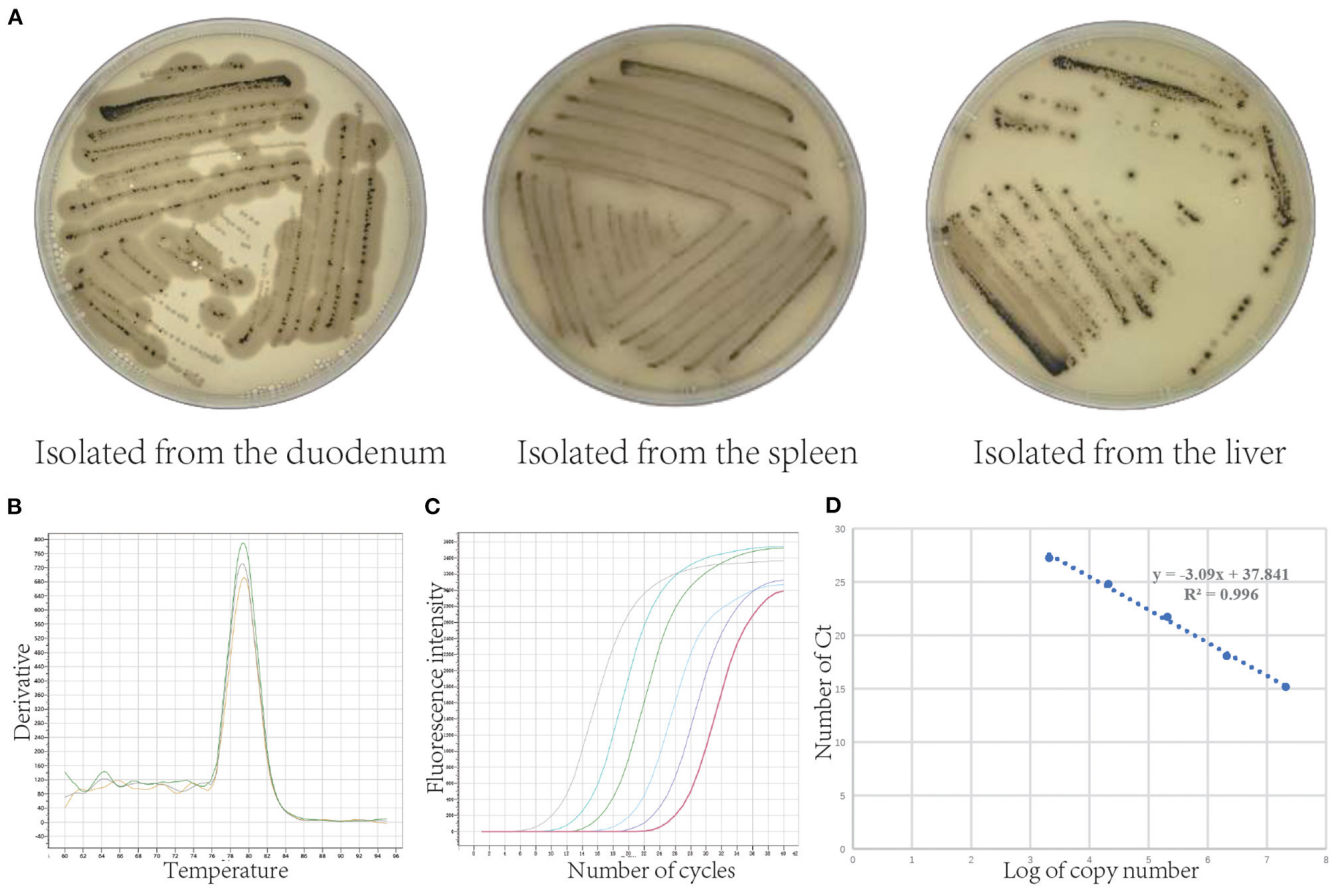
Isolation of *Clostridium perfringens* and its absolute quantification standard curve. **(A)** *Clostridium perfringens* isolated from the duodenum, spleen, and liver (*Clostridium perfringens* uses the sulfur in sodium metabisulphite in the TSC medium to produce hydrogen sulfide gas, which reacts with the ferrous ions in ferric ammonium citrate to produce a black ferrous sulfide substance). **(B)** Fluorescence quantitative PCR melting curve. **(C)** Fluorescence quantitative PCR amplification curve. **(D)** Standard curve of *Clostridium perfringens*.

As shown in Figure 2A, *C. perfringens* was separated from the duodenum, spleen, and liver of pigs (flatulence death). We determined that the pathogen is *C. perfringens* (Gene Bank: OL454188) which was 99.79 % identical with *C. perfringens* ATCC13124. Figures 2B,C showed the melting curve and the amplification curve of fluorescence quantitative PCR, which indicated that the process of qRT-PCR is correct. Figure 2D is the standard curve of absolute quantification (*y* = −3.09x + 37.841, *R*^2^ = 0.996). The standard curve was used to calculate the copies/g of *C. perfringens* concentration.

### Growth Performance and Mortality of Mice

**Figure 3:**
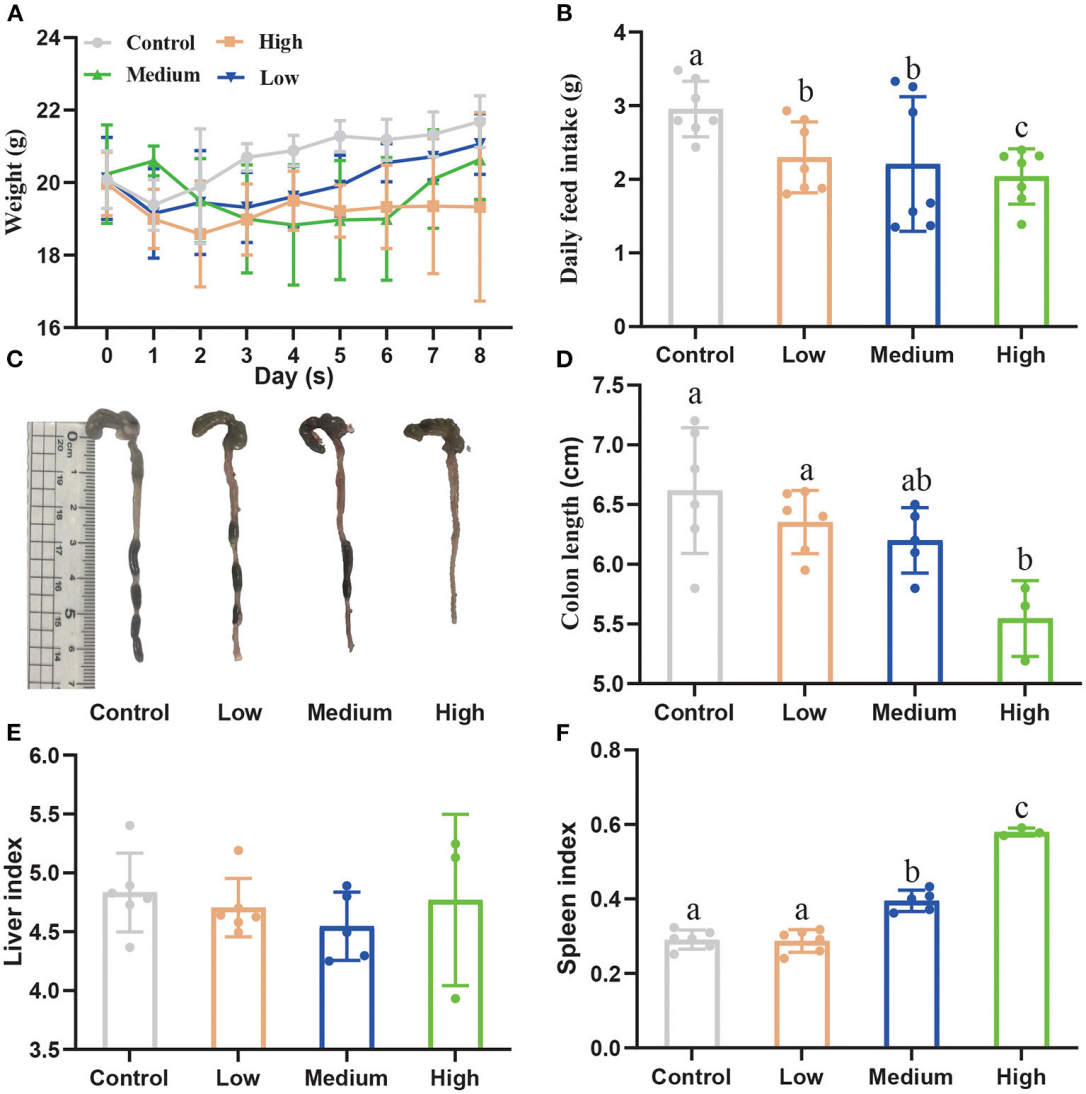
The effect of *Clostridium perfringens* administration on the growth performance of mice. **(A)** Bodyweight (BW) was recorded every day. **(B)** Daily feed intake was recorded among four groups. **(C)** The colon images among four groups. **(D)** The colon length was measured among four groups. **(E)** The liver index. **(F)** The spleen index. ^*a*,*b*,*c*^Means values with dissimilar letters were significantly different (*P* < 0.05). The calculation formula of liver index: liver weight/body weight × 100%. The calculation formula of spleen index: spleen weight/body weight × 100%.

Figure 3A showed the body weights (BW) among four groups during the experimental period. After being challenged with porcine *C. perfringens*, the BW of High group and Medium group decreased from day 1 and 2. Then the BW increased from day 5 to 8 in Medium group, while the BW of High group declined continuously compared with Control group. On day 8, the BW of High group (19.34 ± 1.06 g) significantly decreased (*P* < 0.05) compared with Control group (21.68 ± 0.29 g), whereas Medium group (20.64 ± 0.45 g) and Low group (21.07 ± 0.34 g) had no significant difference (*P* > 0.05) with Control group. Meanwhile, the daily feed intake of all *C. perfringens* treatment groups presented a significant decline (*P* < 0.05) compared with Control group (Figure 3B). Figures 3C,D showed that the colon length dramatically reduced (*P* < 0.05) after high dose treatment, compared with Control group. The liver index (Figure 3E) showed no significant difference (*P* > 0.05) among four groups, while the spleen index (Figure 3F) increased notably (*P* < 0.05) in High and Medium group in contrast to Control group.

**Figure 4:**
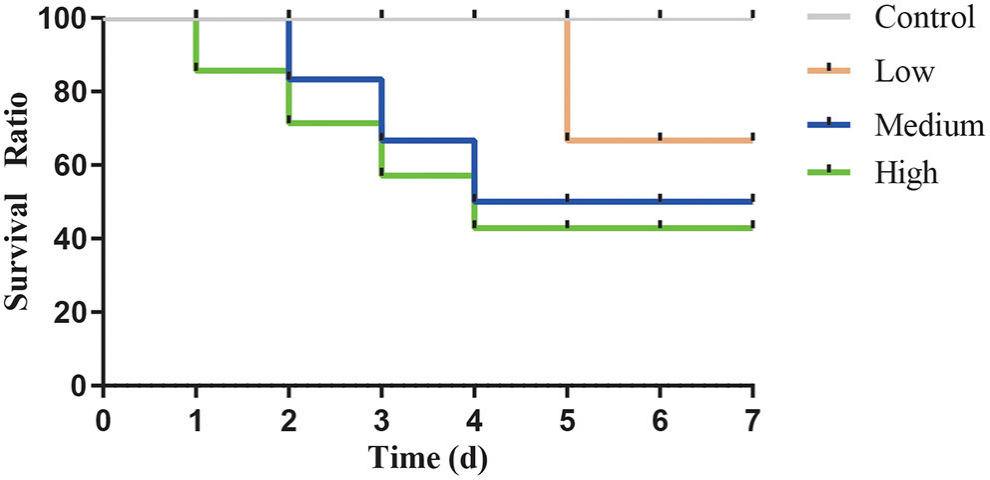
The survival curve after intragastric administration of *Clostridium perfringens*.

After gavage, all groups except Control group displayed the clinical symptoms (Table 3) and the mortality was 12.5% (Low group), 37.5% (Medium group), and 50% (High group), respectively. As the dose rose, Figure 4 showed that the time of death appeared early and the death number further increased.

**Table 3:** The time of clinical symptoms and the number of deaths after gavage of different doses.

**Group**	**Number of bacteria** **inoculated (CFU/mL)**	**Time to onsets** **of symptoms (h)**	**Number of** **deaths**
Control	0	N	0
Low	8.0 × 10^7^	96	1
Medium	4.0 × 10^8^	48	3
High	2.0 × 10^9^	24	4

### *Clostridium perfringens* Stimulated the Injury of Intestines

**Figure 5:**
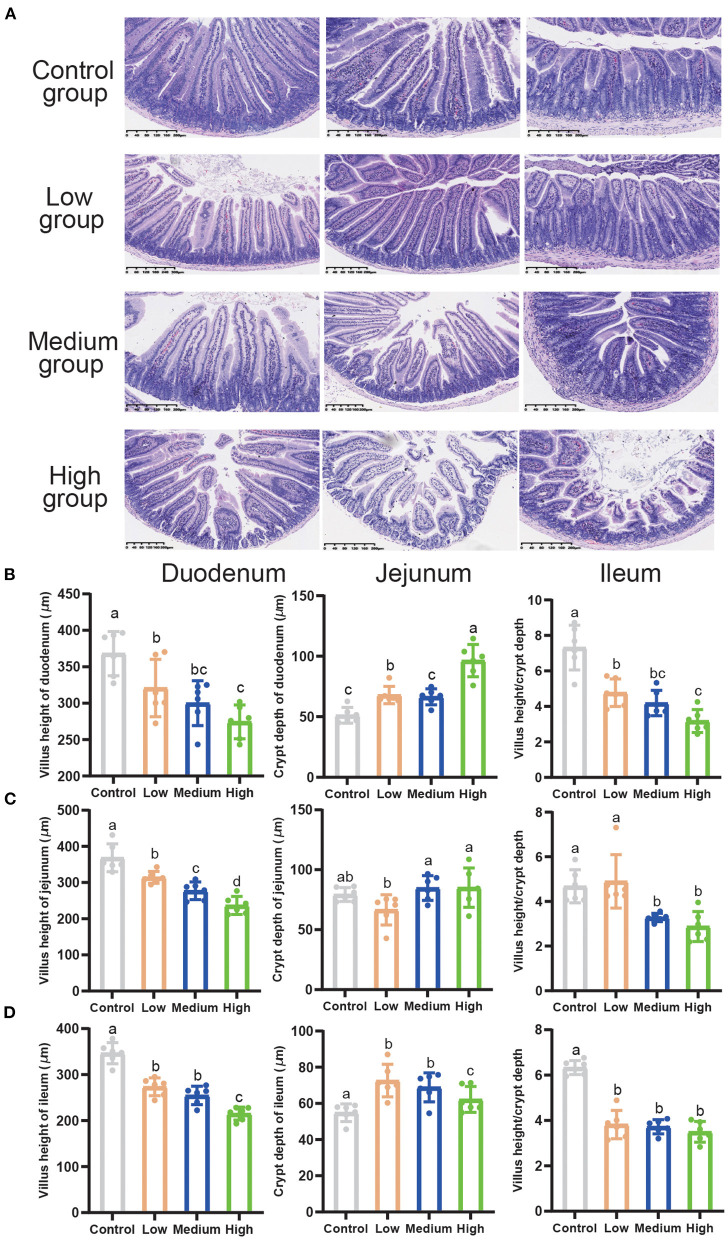
The effects of *Clostridium perfringens* treatment on the intestines of mice. **(A)** Duodenum, jejunum, ileum, liver, and spleen were stained with hematoxylin and eosin (H&E) (bars = 330 μm). **(B)** Villus height of duodenum, crypt depth of duodenum, and villus height/crypt depth. **(C)** Villus height of jejunum, crypt depth of jejunum, and villus height/crypt depth. **(D)** Villus height of ileum, crypt depth of ileum, and villus height/crypt depth. All the values contained six repetitions. ^*a*,*b*,*c*,*d*^Means values with dissimilar letters were significantly different (*P* < 0.05).

Compared to the Control group, the High group exhibited extremely discontinuous brush edges and blunt villus in duodenum, jejunum, and ileum, while Low group had a litter influence which was induced by *C. perfringens* (Figure 5A). Figures 5B–D presented the measured values of intestinal villus height, crypt depth, and the ratio of villus height/crypt depth. Compared with the other three groups, the villus height of the duodenum, jejunum, and ileum in High group significantly decreased (*P* < 0.05), while the crypt depth dramatically increased (*P* < 0.05). Furthermore, we found that the villus height of both Low and Medium groups significantly decreased (*P* < 0.05). Compared to Control group, villus height/crypt depth decreased (*P* < 0.05) in High group. However, the villus height/crypt depth of jejunum in Low group had no difference with Control group.

### Effect of *Clostridium perfringens* Treatment on Inflammatory Cytokines and Immunoglobulin of Mice

**Figure 6:**
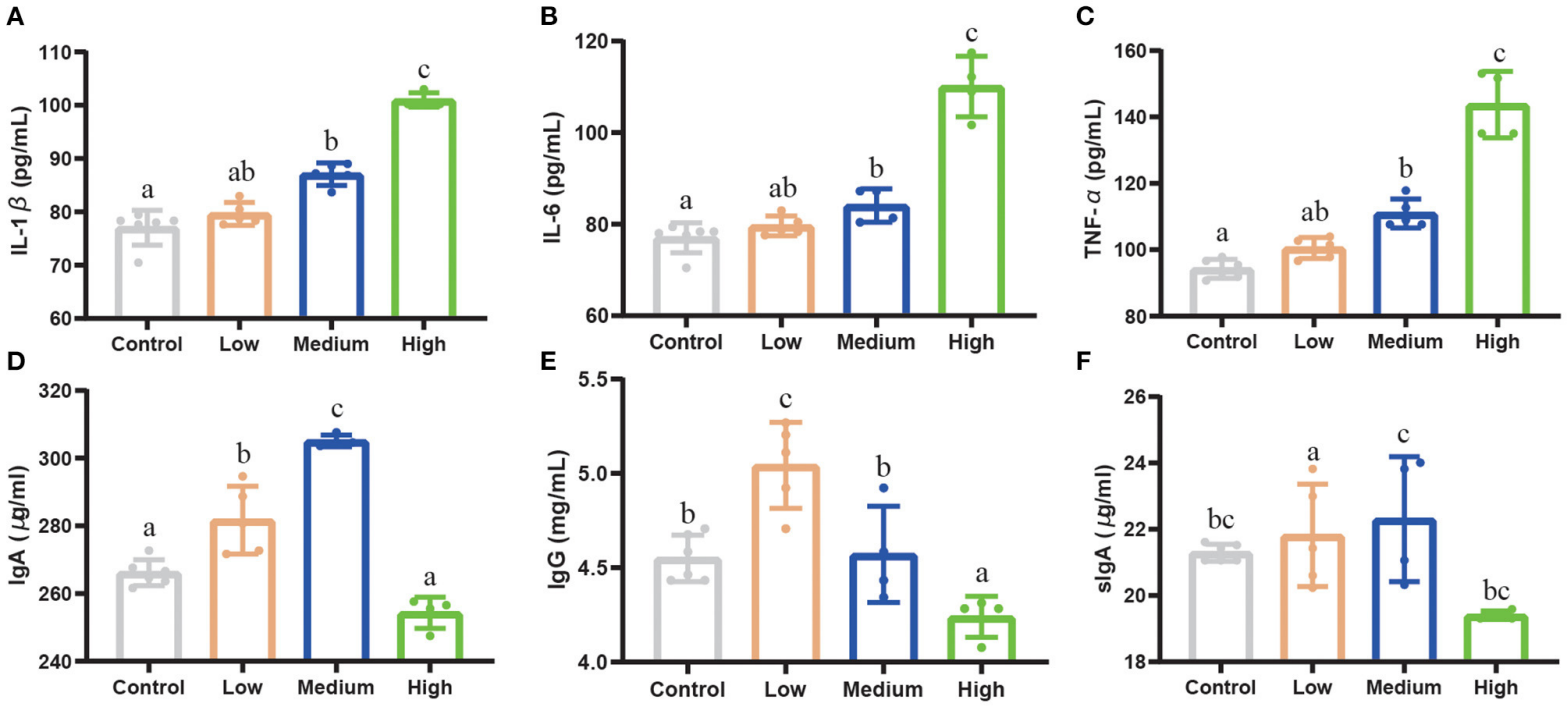
The effect of *Clostridium perfringens* treatment on inflammatory cytokines and immunoglobulin of mice. **(A)** IL-1β. **(B)** IL-6. **(C)** TNF-α. **(D)** IgA. **(E)** IgG. **(F)** sIgA. Results are presented as mean ± SD. ^*a*,*b*,*c*^Means values with dissimilar letters were significantly different (*P* < 0.05).

High and Medium dose *C. perfringens* infection significantly increased (*P* < 0.05) the concentration of IL-1β, IL-6, and TNF-α compared with Control group (Figures 6A–C). However, the Low group had no difference (*P* > 0.05) compared with Control group. For IgA, IgG, and sIgA concentrations (Figures 6D–F), the results were opposite to pro-inflammatory factors. Compared to Control group, the High group did not show a difference (*P* >0.05) in the concentrations of IgA, IgG, and sIgA, while the Medium and Low dose group showed a remarkable upward trend (*P* < 0.05).

### Tight Junctions and Apoptosis Related Genes Expression

**Figure 7:**
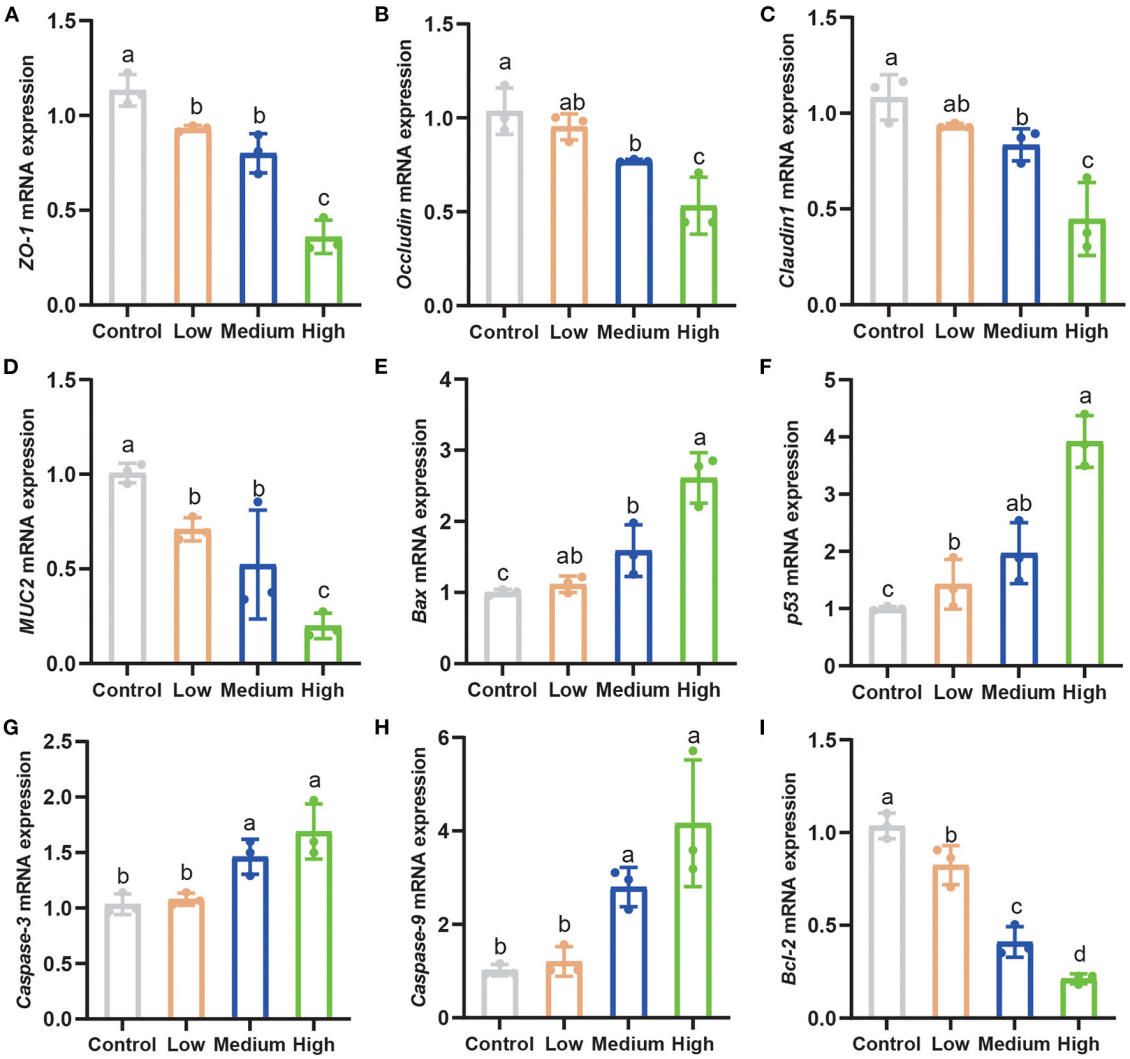
*Clostridium perfringens* facilitated apoptosis and weakened tight junctions related genes expression. **(A–C)** The tight junctions related genes expression in the jejunum of mice. **(D–I)** The apoptosis-related gene expression in the jejunum. Results are presented as mean ± SD. ^*a,b,c,d*^Means values with dissimilar letters were significantly different (*P* < 0.05).

The tight junction-related genes (*ZO-1, Occludin, Claudin1*, and *MUC2*) in the jejunum down-regulated significantly (*P* < 0.05) in all *C. perfringens* infection groups compared to Control group (Figures 7A–D). Furthermore, with the dose rising, the gene expression of tight junctions decreased seriously. Pro-apoptosis genes including *Bax, p53, Caspase-3*, and *Caspase-9* elevated dramatically (*P* < 0.05) in the High group and Medium group in contrast to Control group and Low group (Figures 7E–H). High group enhanced the pro-apoptosis genes' expression stronger than other groups. Similarly, the anti-apoptosis gene (*Bcl-2*) saw a notable decrease (*P* < 0.05) among three *C. perfringens* stimulated groups compared with Control group (Figure 7I).

### Ileal and Cecal *Clostridium perfringens* Enumeration

**Figure 8:**
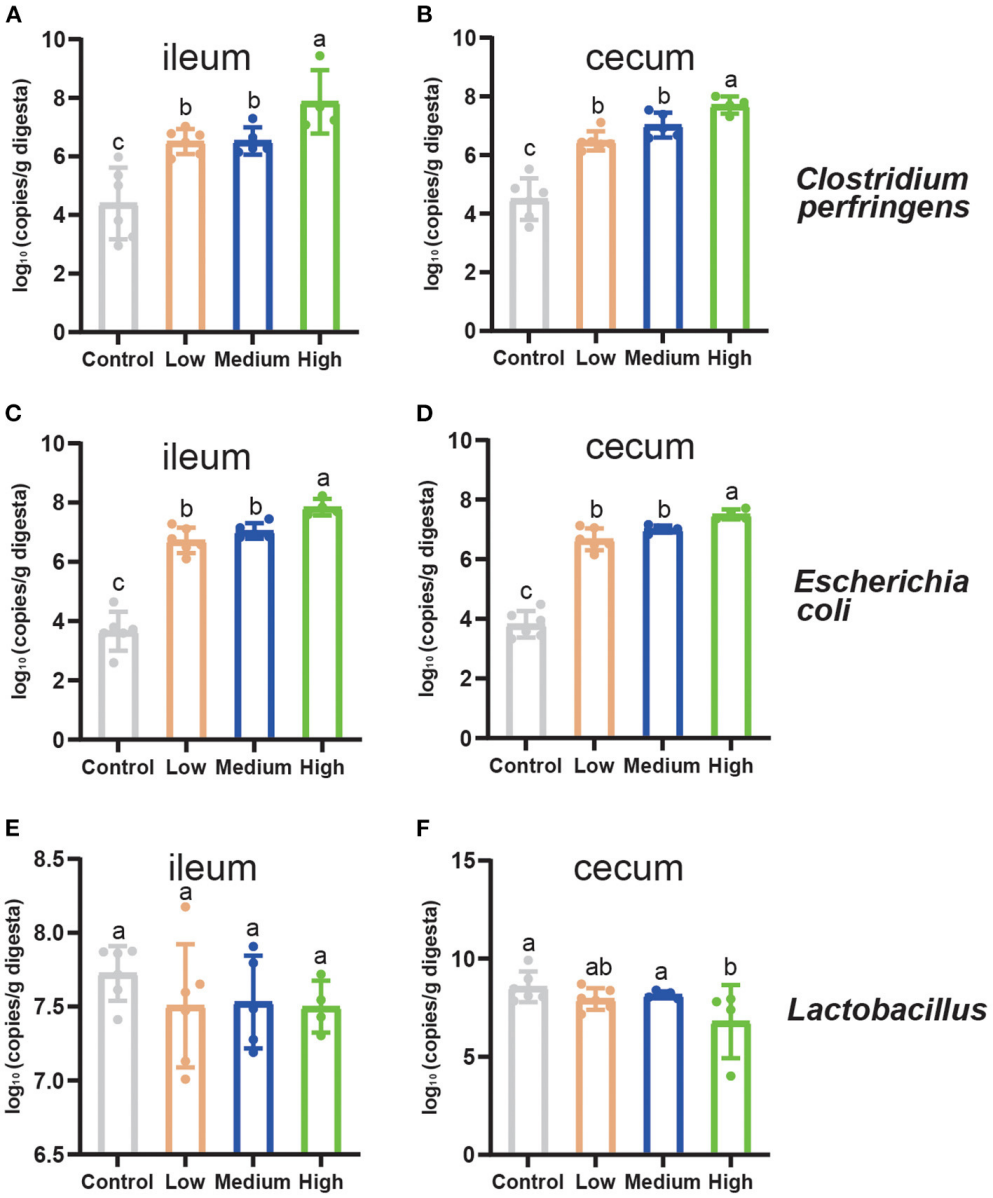
The quantitation of intestinal microbiota of mice on d 8. **(A)** The population of *Clostridium perfringens* in the ileum. **(B)** The population of *Clostridium perfringens* in the cecum. **(C)** The population of *Escherichia coli* in the ileum. **(D)** The population of *Escherichia coli* in the cecum. **(E)** The population of *Lactobacillus* in the ileum. **(F)** The population of *Lactobacillus* in the cecum. Tight junctions related genes expression in the jejunum of mice. Results are presented as mean ± SD (The data were presented as log_10_ gene copies/g of intestinal digesta). ^*a*,*b*,*c*^Means values with dissimilar letters were significantly different (*P* < 0.05).

Figure 8 showed the results of the *C. perfringens, Escherichia coli*, and *Lactobacillus* enumeration in two different segments of small intestine in mice. Compared to Control group, the population of *C. perfringens* and *Escherichia* coli increased significantly in cecum and ileum (*P* < 0.05) by *C. perfringens* challenge. The genes copies of *C. perfringens* and *Escherichia coli* enriched remarkably (*P* < 0.05) in High group compared to Low and Medium group. Meanwhile, the population of *Lactobacillus* remained steady (*P* > 0.05) in ileum among four groups, while the *Lactobacillus* of cecal digesta decreased dramatically (*P* < 0.05) in High group in contrast to Control group.

## Discussion

Enteric disease that occurrs in suckling and finishing pigs is usually caused by *C. perfringens* type A or type C, and this pathogen has been linked to necrotizing enterocolitis and villous atrophy in pig's intestines. Lesions are always most severe in the small intestine, especially the jejunum and ileum (16). Isolating the pathogenic *C. perfringens* and establishing infection animal model is an essential step to prevent and treat clinical diseases in the animal production process. In this study, we aimed to investigate the suitable concentration of *C. perfringens* to establish a mouse model.

We used the same method as Kareem Rashid Rumah et al. to isolate the type B P. aeruginosa strain by sandwiching fecal samples in TSC agar (30). Similarly, Ruofan Wang et.al and Rodrigo et.al had successfully accessed the sequencing biotechnology to determine the genera of isolation bacteria from soil (*C. perfringens* type A) and cat (*C. perfringens* type A), respectively (31, 32).

For the animal experiment, the growth performance of three different concentrations of *C. perfringens* were different from Control group, which indicated that *C. perfringens* treatment can influence the growth status of mice. Furthermore, with the dose increasing, the daily feed intake and colon length decreased significantly (*P* < 0.05) compared with Control group. However, spleen index increased significantly (*P* < 0.05) in Medium and High group. The main reason for these results is the damage to the intestinal segments of the mice and a series of immune responses caused by C. perfringens infection, resulting in reduced food intake and swelling of the organs. The survival curve illustrated that the LD50 dose is High dose (2.0 × 10^9^ CFU/mL). For oral challenge mouse model studies, Mariano E.Fernandez-Miyakawa et.al used *C. perfringens* type D to study the lethality (7 of 10 type D isolates were lethal) and they provided the lethality of the seven type D isolates varying from 14 to 100% (15). Similarly, Uzal et al. (17) found that intragastric or intraduoden challenge in mice can trigger respiratory distress and abdominal distension, and they found that, when inoculated into mice by intragastric gavage, 7 of 14 type C isolates were lethal, while when inoculated intraduodenally, all strains were lethal. These results indicated that pathogens have a different ability to produce toxins and LD50 were unequal even in the same type of *C. perfringens*. Our study indicated that this porcine *C. perfringens* type A isolated from our laboratory is infectious and toxic and it is suitable for building the mouse model.

It is known that the physical barrier is the first line of defense against pathogens invading into the intestine, and gut health level is related to villus atrophy and crypt hyperplasia degree (33). Our previous study indicated that *C. perfringens* infection can trigger serious villous atrophy and intestinal morphology disruption (21). This study also demonstrated that High dose *C. perfringens* inoculation impaired the development of the small intestinal morphology, weakened the villus morphology, decreased the ratio of villus height/crypt depth, and triggered an irreparable effect on the crypt depth in the small intestine. Some studies in broilers presented the same results in intestinal morphology (34–37). Other studies found that the *C. perfringens* enterotoxin (CPE), which is the main toxin produced by *C. perfringens* type A, displayed a dose-dependent effect and CPE can induce the intestinal injury (38–40). These studies focused on the toxaemic outcome of toxin absorption from the intestine into the circulation, and they did not adequately examine the specific changes that occur during *C. perfringens* infection. In the present study, we used a mouse model by oral gavage to understand the pathogenesis of porcine gas-producing podoconiosis. Our results suggested that high doses of *C. perfringens* have a negative effect on intestinal damage and the High dose (2.0 × 10^9^ CFU/mL) could construct the mouse model, too. One possible reason is that the high dose of *C. perfringens* causes an imbalance in the intestinal flora and *C. perfringens* can overgrow and induce inflammation in the intestine of mice, leading to a series of negative effects (weight loss, decreased expression of tight junction proteins, intestinal morphological damage, etc.).

To some extent, the serum inflammatory cytokines and immunoglobulin can reflect the physiological and immune status of mice after *C. perfringens* infection. Serum IL-1β, IL-6, and TNF-α are indicators of pro-inflammatory reaction, which can be observed in the *C. perfringens* infected animals (41–43). IgA can mediate a variety of protective functions and also has both anti-inflammatory and pro-inflammatory effects (44). Similar studies report that IgA deficiency can induce the inflammation, specifically in the ileum. IgG is the major serum immunoglobulin involved in mediating a protective inflammatory response (45). Secretory immunoglobulin A (sIgA) is the most abundant colonic antibody antigen and it can improve the immune status of animals (46). Following infection with opportunistic pathogens, cytokine expression of IL-1β, IL-6, and TNF-α in the mucosa and serum increased significantly (*P* < 0.05), which resulted in decreased immunity (47, 48). Our results demonstrated that with the infectious dose increasing, the pro-inflammatory cytokines rose significantly (*P* < 0.05) compared with Control group, especially in High group. IgA, IgG, and fecal sIgA increased (*P* < 0.05) significantly in Low group and Medium group, which indicated that mild pathogens infection can stimulate the immunoglobulin secretion. However, the High dose may lower the immune status of an animal so that the host could not respond to pathogen invasion adequately and accurately.

Tight junctions play a vital role in separating tissue compartments and maintaining cellular polarity (49). The core complex is composed of ZOs, Occludin, and Claudin family members, which connect the intestinal epithelial cells and regulate paracellular permeability (50). Meanwhile, the MUC2 is the structural component of the intestinal epithelium mucus layer and its expression is lowered in inflammatory bowel disease (51). Epithelial damage, in particular the tight junction proteins and mucins affecting the protective properties, likely induces the inflammation (52). The tight junctions related genes and MUC2 gene expression (Figures 7A–D) in the jejunum of mice decreased significantly (*P* < 0.05) in Medium and High group. Our results demonstrated that the adequate concentrations of porcine *C. perfringens* can injure the tight junctions seriously in the jejunum of mice. Additionally, apoptosis is critical for the normal development and function of multicellular organisms, which are initially activated by the imbalance proteins expression, such as pro-apoptosis protein Bax and anti-apoptosis protein Bcl-2 (53). After the imbalance occurs, the apoptosis process began, and the main characteristic is the release of cytochrome c from the mitochondria. Then Caspase-9 and downstream executioner Caspases-3 are activated, thereby initiating cell apoptosis (54). In addition, p53 protein can induce apoptosis by inhibiting the expression of anti-apoptosis gene (survivin) and promoting the pro-apoptosis (Bax), thereby triggering apoptosis through caspase-dependent pathway (55). Using a mouse model, *C. perfringens* enterotoxin was shown to cause intestinal caspase-3 activation in a dose- and time-dependent manner (38). Using the broiler model which was infected by *C. perfringens*, the pro-apoptosis related genes also showed a significant increase (*P* < 0.05) and a dramatic decrease (*P* < 0.05) in anti-apoptosis related genes (56). Our previous study presented the same results, C57B/L mice infected with *C. perfringens* ATCC 13124 showed a significant decrease in jejunal Bcl-2 gene expression and a significant increase in Bax, p53, Caspase-3 and Caspase-9 gene expression (21). In the present study, we found that the High group can induce a stronger apoptosis signal compared with other groups.

Many bacteria have been shown to coexist with *C. perfringens* when the infection occurs, including *Escherichia coli*. *Escherichia coli* is a major enteric pathogen causing intestinal diseases (57). Gao et.al exerted the same method and established the standard curve of *Escherichia coli* K88 to detect it in the small intestinal contents of piglets (58). Meanwhile, Zhui Li et.al used a similar method to detect the *C. perfringens* type A content in ileum and cecum of broilers (25). All these studies found that the aimed bacterial content in infection group increased significantly (*P* < 0.05) compared to control group. As with *C. perfringens* infection, the population of *Escherichia coli* increased significantly, which might influence the intestine to counteract the serum endotoxin secreted by *C. perfringens* (25). *Lactobacillus* species have been known as probiotics, which can produce bacteriostatic bacteriocin-like compound and acids, including lactic acid (59). Other studies reported that *Lactobacillus* can prevent the proliferation of pathogenic bacteria and regulate the intestinal flora (60, 61). In addition, *Lactobacillus* plays an important role in maintaining the gut health of animal (62) and modulating immunity (63). In the present study, *C. perfringens* challenge significantly increased (*P* < 0.05) the population of *C. perfringens* and *Escherichia coli* in the ileum and cecum, which are consistent with previous studies (24, 64). However, compared to Control group, the *Lactobacillus* of cecum significantly decreased (*P* < 0.05). Other previous studies (24, 64) reported that, after being challenged with *C. perfringens*, the *Lactobacillus* of cecum also increased, while our study showed the opposite. One possible reason to explain this is the high dose of *C. perfringens* could alter the balance of microbial community in mice, while low and medium doses might alter the microbiota composition to some degree or not at all.

## Conclusion

In summary, the present study evaluated the effects of different porcine *C. perfringens* dose (High: 2.0 × 10^9^ CFU/mL, Medium: 4.0 × 10^8^ CFU/mL, Low: 8.0 × 10^7^ CFU/mL) treatments in mice by oral gavage. The High group meet the requirements for constructing mouse models by reducing growth performance, damaging intestinal morphology, reducing immune status, promoting apoptosis, and increasing the number of pathogens. Furthermore, these results provided an experimental and theoretical basis for the construction of porcine *C. perfringens* infection model in mice.

## Data Availability Statement

The datasets presented in this study can be found in online repositories. The names of the repository/repositories and accession number(s) can be found in the article/supplementary material.

## Ethics Statement

The animal study was reviewed and approved by Institutional Animal Care and Use Committee at Zhejiang University.

## Author Contributions

ZJ: conceptualization, methodology, investigation, and writing original draft. WS, WL, CW, and SD: investigation and visualization. YZ and TG: formal analysis and visualization. XW: writing–review and editing. MJ, ZL, and YW: resources, writing–review, editing, and supervision. All authors contributed to the article and approved the submitted version.

## Funding

The authors thank the specialized research fund from Major Science and Technology Projects of Zhejiang (2021C02008, 2022C02043, and CTZB-2020080127), China Agriculture Research System of MOF and MARA (CARS-35), National Center of Technology Innovation for Pigs.

## Conflict of Interest

The authors declare that the research was conducted in the absence of any commercial or financial relationships that could be construed as a potential conflict of interest.

## Publisher's Note

All claims expressed in this article are solely those of the authors and do not necessarily represent those of their affiliated organizations, or those of the publisher, the editors and the reviewers. Any product that may be evaluated in this article, or claim that may be made by its manufacturer, is not guaranteed or endorsed by the publisher.
